# Therapeutic evidence of umbilical cord-derived mesenchymal stem cell transplantation for cerebral palsy: a randomized, controlled trial

**DOI:** 10.1186/s13287-019-1545-x

**Published:** 2020-02-03

**Authors:** Jiaowei Gu, Li Huang, Che Zhang, Yong Wang, Ruibo Zhang, Ziliang Tu, Hengdong Wang, Xihui Zhou, Zhousheng Xiao, Zegan Liu, Xiang Hu, Zunchen Ke, Dabin Wang, Li Liu

**Affiliations:** 1 Department of Neonatology, The First Affiliated Hospital of Xi’an Jiaotong University, No.277 West Yanta Road, Xi’an, 710061 Shaanxi People’s Republic of China; 2 Affiliated Taihe Hospital of Hubei University of Medicine, No. 32 Southern Renmin Road, Shiyan, 422000 Hubei People’s Republic of China; 3 Department of Medicine, University of Tennessee Health Science Center, Memphis, TN 38163 USA; 4 Shenzhen Beike Biotechnology Co., Ltd, No. 18 Keyuan Road, Hi-Tech Industrial Park South Area, Shenzhen, 518057 People’s Republic of China; 5 Shiyan City Disabled Persons’ Federation, No. 12 Beijing Road, Shiyan, 422000 Hubei People’s Republic of China

**Keywords:** Mesenchymal stem cells, Umbilical cord, Cerebral palsy, Clinical trial, Cell transplantation, Activities of daily living

## Abstract

**Background:**

Cerebral palsy (CP) is a syndrome of childhood movement and posture disorders. Clinical evidence is still limited and sometimes inconclusive about the benefits of human umbilical cord mesenchymal stem cells (hUC-MSCs) for CP. We conducted a randomized trial to evaluate the safety and efficacy of hUC-MSC transplantation concomitant with rehabilitation in patients with CP.

**Methods:**

Eligible patients were allocated into the hUC-MSC group and control group. In addition to rehabilitation, the patients in the hUC-MSC group received four transfusions of hUC-MSCs intravenously, while the control group received a placebo. Adverse events (AEs) were collected for safety evaluation in the 12-month follow-up phase. Primary endpoints were assessed as activities of daily living (ADL), comprehensive function assessment (CFA), and gross motor function measure (GMFM) scales. In addition, cerebral metabolic activity was detected by ^18^F-FDG-PET/CT to explore the possible mechanism of the therapeutic effects. Primary endpoint data were analyzed by ANOVA using SPSS version 20.0.

**Results:**

Forty patients were enrolled, and 1 patient withdrew informed consent. Therefore, 39 patients received treatments and completed the scheduled assessments. No significant difference was shown between the 2 groups in AE incidence. Additionally, significant improvements in ADL, CFA, and GMFM were observed in the hUC-MSC group compared with the control group. In addition, the standard uptake value of ^18^F-FDG was markedly increased in 3 out of 5 patients from the hUC-MSC group at 12 months after transplantation.

**Conclusions:**

Our clinical data showed that hUC-MSC transplantation was safe and effective at improving the gross motor and comprehensive function of children with CP when combined with rehabilitation. Recovery of cerebral metabolic activity might play an essential role in the improvements in brain function in patients with CP. The therapeutic window, transfusion route, and dosage in our study were considerable for reference in clinical application.

**Trial registration:**

Chictr.org.cn, ChiCTR1800016554. Registered 08 June 2018—retrospectively registered. The public title was “Randomized trial of umbilical cord-derived mesenchymal stem cells for cerebral palsy.”

## Background

Cerebral palsy (CP) is a syndrome of movement and posture disorders caused by non-progressive impairments in the developing brain [1]. It is typically accompanied by associated disturbances, such as cognition difficulties, hearing or visual impairments, and epilepsy [2]. Therefore, CP is considered a main cause of disabilities in childhood [3]. The overall prevalence of CP was reported as 2.11 per 1000 live births [4]. Thus, CP has become a growing public health concern worldwide due to its impact on the quality of the population and heavy burden on related families [5] and financial resources [6]. Although the pathogenesis is still unclear, multiple risk factors were identified to facilitate early diagnosis of CP, including neonatal encephalopathy, intrauterine infection, preterm delivery, congenital anomalies, and abnormal fetal inflammatory response [7]. Additionally, neonatal magnetic resonance imaging (MRI) was suggested as a sensitive and predictive tool for CP diagnosis [8]. Typically, the therapeutic strategies require multidisciplinary cooperation due to the complex clinical manifestations of CP. Current supportive treatments rely on neurotrophic medications, orthopedic surgery, rehabilitation, and hyperbaric oxygen treatments [9]. However, the therapeutic efficacy is limited since none of the treatments targets cerebral injury [10]. Therefore, novel therapeutic options are needed to further promote the physical function and quality of life of patients.

Recently, stem cell intervention has shed light on the treatment strategy targeting a cure [11]. Human umbilical cord mesenchymal stem cells (hUC-MSCs) are attractive because of their easy accessibility, low immunogenicity, and immunosuppressive potential compared with other types of stem cells [12]. Functional improvements with alleviation of brain lesions were detected after hUC-MSC transplantation in a series of animal model studies [13–15]. In clinical case reports, gross motor and cognitive functions were improved after hUC-MSC [16] and bone marrow mononuclear cell transplantation [17]. In addition, gross motor function classification scores were promoted significantly after human embryonic stem cell treatment in a retrospective cohort analysis [18]. Although preliminary benefits were shown in these studies, further therapeutic evidence is demanded in randomized clinical trials for systemic evaluation. To date, 27 clinical trials have been registered on ClinicalTrials.gov for cell therapy in CP. hUC-MSCs were given to patients in four trials. More safety and therapeutic evidence is needed to fill the gap from bench to bedside.

In China, rehabilitation is a common intervention for CP. However, it is still unknown whether the treatment efficacy of rehabilitation could be further promoted by hUC-MSC transplantation. In addition, little is known about the mechanism of functional improvement after stem cell treatments in CP. To assess the therapeutic potential of hUC-MSC transplantation in patients with CP, we conducted a randomized, double-blinded, placebo-controlled trial. In addition, cerebral metabolic activity was detected using ^18^F-FDG-PET/CT to advance the understanding of the underlying mechanism.

## Materials

### Study design and procedure

**Fig. 1:**
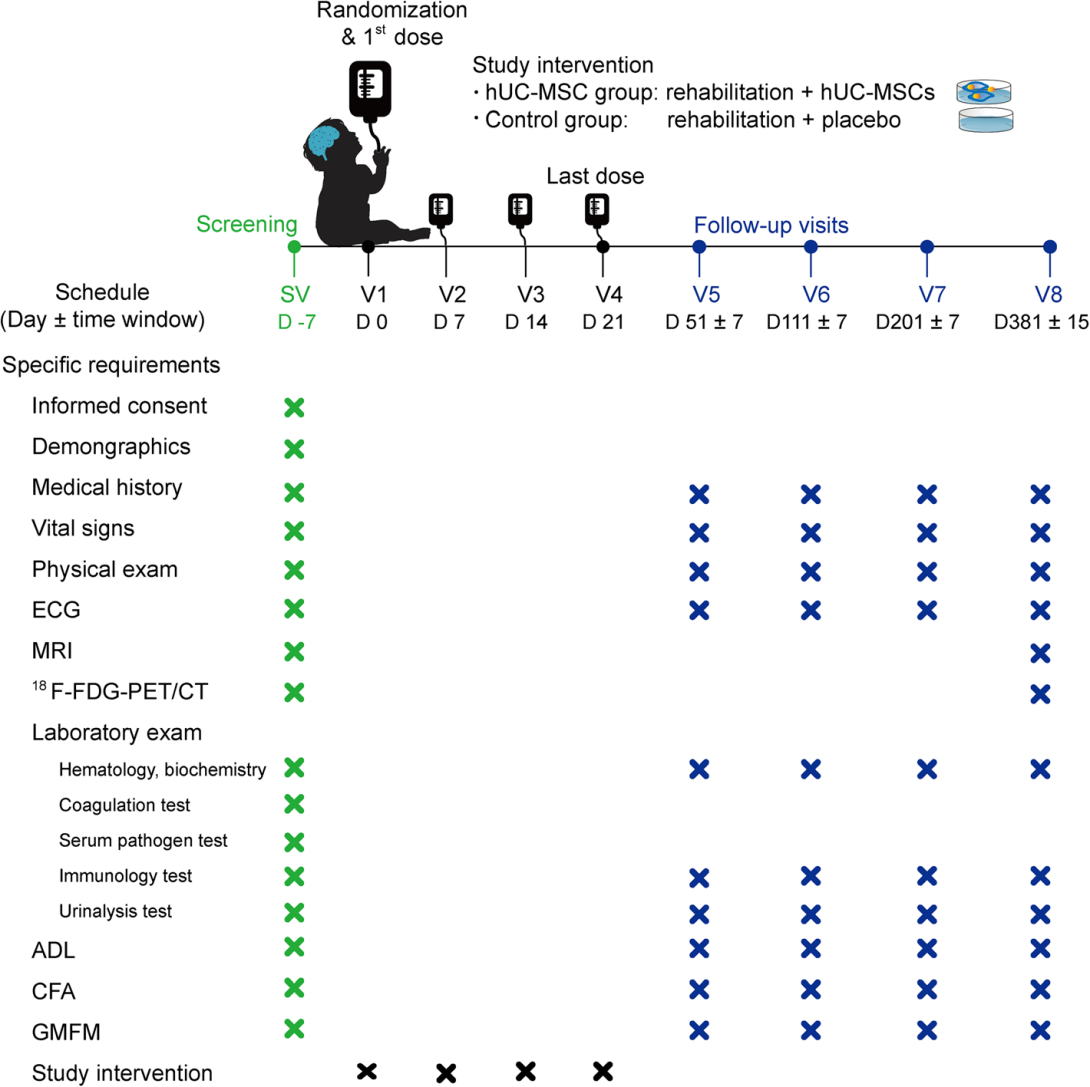
Flow chart for the study procedure. The patients received four transfusions after randomization and completed four scheduled visits at 1 month (51 ± 7 days), 3 months (111 ± 7 days), 6 months (201 ± 7 days), and 12 months (381 ± 15 days) after the last dose. ADL, activities of daily living; CFA, comprehensive function assessment; ECG, electrocardiogram; GMFM, gross motor function measure; hUC-MSCs, human umbilical cord blood mesenchymal stem cells; MRI, magnetic resonance imaging; ^18^F-FDG PET/CT, ^18^F-fluorodeoxyglucose positron emission tomography/computed tomography

This trial was designed as a parallel randomized, double-blinded, placebo-controlled study to evaluate the efficacy and safety of hUC-MSC transplantation concomitant with rehabilitation in patients with CP. Baseline examinations were performed within 7 days prior to randomization, including physical exams, laboratory tests, electrocardiogram, and MRI. The main laboratory tests included tests for hematology, biochemistry, blood coagulation, serum pathogens, immunology, and urinalysis. Eligible patients were randomly allocated into two groups at a 1:1 ratio and received hUC-MSCs or placebo intervention as assignments. Safety was evaluated with the adverse event (AE) incidence. Primary efficacy assessments were performed at 1, 3, 6, and 12 months after the last dose (Fig. 1), including activities of daily living (ADL) [19], comprehensive function assessment (CFA) [20], and gross motor function measure (GMFM) [21]. Moreover, cerebral metabolic activity was detected by ^18^F-fluorodeoxyglucose positron emission tomography/computed tomography (^18^F-FDG-PET/CT) at baseline and 12 months after the last dose for exploratory research.

### Study approval

Ethical approval was available before study-specific procedures from the institutional review board (IRB) of Affiliated Taihe Hospital of Hubei University of Medicine (ethical approval no. 20140801). Written informed consent was obtained from patients and their guardians prior to screening. In addition, patients were identified by numbers instead of their names for the sake of privacy protection. This trial was registered on the World Health Organization international clinical trials registered organization platform, China Chictr.org.cn (ChiCTR1800016554). The full trial protocol (version 1.1, version date 20140724) could be accessed from this platform.

### Study population

The patients were recruited from Affiliated Taihe Hospital of Hubei University of Medicine. Eligibility criteria for candidates included a diagnosis of CP. In addition, candidates aged between 2 and 12 years could be included in the study. Candidates had to demonstrate their willingness by signing informed consent forms and complying with all the study procedures, including treatment schedule, examination plans, and performance scale evaluation. Criteria for diagnosis of CP [22, 23] were as follows: (a) permanent motor and posture disorders caused by an insult to the developing infant brain; (b) non-progressive disturbances; (c) delayed developmental presentation in infancy; (d) could be accompanied by cognitive impairment, disturbance of sensation, epilepsy, and musculoskeletal problems; and (e) exclusion of motor defects caused by progressive diseases and transient developmental delays in normal cases.

Candidates were excluded with one of the following conditions: (a) grand mal seizure within 15 days or seizure attack within 24 h prior to treatment, (b) congenital heart disease, (c) any known genetic or immunological disease, (d) coagulation disorder, (e) serious liver or renal dysfunction, (f) malignancy history, (g) serious allergy history or known allergy to more than two kinds of food or medications, (h) active serious infection, (i) participation in other clinical trials within 3 months prior to screening, and (j) any other concerns that hampered the compliance or safety as judged by the investigator.

### Sample size, randomization, and blinded procedure

Based on our pilot studies, the sample size was calculated based on the assumption that clinical benefit was achieved for 60% of patients in the hUC-MSC group and 10% of patients in the control group (*α* = 0.05, *β* = 0.10, allocation ratio 1:1, by two-tailed tests). Thus, at least 14 subjects were required for each group. Therefore, 20 participants were planned for each group considering the withdrawal rate.

A randomized block design was utilized with 10 participants in a block. The eligible patients were assigned to one of two groups at a 1:1 ratio according to a random number table, which was generated by a biostatistician with SAS 9.0.

To facilitate the blinding procedure, randomization information was sealed in opaque envelopes and delivered to the authorized investigator according to the sequence of screening. The patients and their families, as well as the investigators, were all blinded to the grouping information. In addition, injection preparation and administration were completed by separate staff in different departments. The staff was blinded to patient information when preparing hUC-MSC injection or placebo in the Pharmacy Intravenous Admixture Service (PIVAS). The study nurse was blinded to the treatment information when completing administration to patients in the Cell Treatment Center. Placebo could not be distinguished from hUC-MSC injection by their appearance. According to the unblinding process, treatment information would be revealed only in emergency cases for safety management. All the investigators and study staff received sufficient training in GCP and the study protocol before study initiation.

### hUC-MSC source

Allogeneic hUC-MSCs were purchased from Shenzhen Beike Biotechnology Company Co., Ltd. hUC-MSCs were collected from umbilical cord (UC) Wharton’s jelly (WJ). In brief, UC samples were obtained from healthy puerperal women after informed consent was obtained. Eligible donors passed physical exams with negative results in tests for syphilis, human immunodeficiency virus, and hepatitis virus, according to the donor requirements of the American Association of Blood Banks [24]. UCs were cut into pieces after washing with phosphate-buffered saline three times. Then, WJ was exposed for incubation after removing vessels from pieces of UC. After isolation from WJ, harvested cells were cultivated in serum-free Dulbecco’s modified Eagle’s medium (DMEM) supplemented with cytokines. Then, cell surface marker analysis was performed on cells at passage 4 using a flow cytometer (FACSCalibur, BD Biosciences, San Jose, CA, USA). Markers with a positive percentage less than 2% were considered negative for expression. Thus, the harvested cells were tested for high expression of MSC-specific surface markers (> 95%) and negative expression of hematopoietic stem cell-specific markers (Table 1). The harvested hUC-MSCs were stored at − 196 °C under cryopreservation conditions. Quality tests were conducted on each batch of cell products before clinical usage according to the International Society for Cellular Therapy standards [25] (Table 1).

**Table 1:** Characteristics of human umbilical cord mesenchymal stem cells for transplantation

Quality parameters	Results (*n* = 15^A^)
Cell surface markers	
CD14	Negative
CD34	Negative
CD45	Negative
CD79a/CD19	Negative
HLA-DR	Negative
CD73, mean ± SD (%)	97.51 ± 0.41
CD90, mean ± SD (%)	98.88 ± 0.52
CD105, mean ± SD (%)	95.52 ± 0.42
Viability rate, mean ± SD (%)	98.50 ± 0.26
Cell count (cell/ml)	(5.00 ± 0.50) × 10^7^
Pathogen tests	
Human T cell leukemia virus	Negative
Cytomegalovirus	Negative
Aerobic bacteria	Negative
Anaerobic bacteria	Negative
Fungi	Negative

^A^hUC-MSCs were provided in 15 batches

### Study intervention

Bobath therapy [26, 27] and conductive education [28] were conducted as basic rehabilitation for each patient. Patients received the treatments twice a day and 6 days per week until administrations were completed.

In addition, patients received hUC-MSC transplantation or placebo as assignments. hUC-MSCs were dispersed and diluted in 50 ml of normal saline (NS). A quick check was performed to ensure the appropriate cell count (4.5~5.5 × 10^7^) and viability rate (> 90%) before transplantation. Then, transplantation was completed intravenously in 20~30 min. Placebo was prepared by 50 ml of NS with 1% human serum albumin and was given in the same process as hUC-MSC transplantation. In total, four doses were given with an interval of 7 days (Fig. 1). Immunosuppressants were not given for pre-treatment, based on the safety data in our previous studies.

### Safety assessments

Safety outcomes were evaluated by AE incidences. AEs were defined according to the definition of the International Conference on Harmonization-Good Clinical Practice (ICH-GCP). The terms and grades of AEs were in accordance with the Common Terminology Criteria for Adverse Events (version 5.0) from the US Department of Health and Human Services.

Routine safety monitoring was conducted during scheduled visits. In addition, immunologic tests were performed before and after study intervention for graft rejection monitoring, including tests of immunoglobulin (IgA, IgG, and IgM), complement (C3 and C4), antistreptolysin, rheumatoid factor, and high-sensitivity C-reactive protein. Patient vital signs and consciousness status were monitored closely during transplantation, particularly for fever and leukopenia. MRI was performed using a 3.0-T system (Signa HDX, GE Healthcare, Milwaukee, WI, USA) at baseline and 12 months after treatment. Images were obtained for anatomical analysis in the sequence of axial T1-weighted, T2-weighted, and T2-fluid-attenuated inversion recovery (FLAIR). In addition, other examinations could be conducted in cases of AE handling.

### Efficacy assessments

Primary efficacy analysis was based on the score changes in the ADL, CFA, and GMFM scales at the last visit from baseline. Specifically, a basic ADL scale was used to measure daily self-care ability in the following aspects: personal hygiene, feeding, dressing, toileting, ambulating, using tools, communication, position holding in bed, and walking. The CFA scale was used to evaluate comprehensive function with respect to cognizance, language competence, self-care, motor function, and social adaptability. Moreover, the GMFM-88 scale was used to assess gross motor ability regarding “lying and rolling,” “sitting,” “crawling and kneeling,” “standing,” and “walking, running, and jumping.” These assessments were completed before and after treatments during scheduled visits. The score changes from baseline were compared between groups for efficacy evaluation.

### Exploratory assessment

The standard uptake value (SUV) of ^18^F-fludeoxyglucose (^18^F-FDG) was detected using PET/CT [29] to measure cerebral metabolic activity. This exam was performed at baseline and at the last visit and only for the patients showing their willingness in informed consent forms. Cerebral images were acquired with a PET/CT system (Biograph mCT-S 64, Siemens Healthcare, Erlangen, Germany). ^18^F-FDG was synthesized using a medical cyclotron (HM-10, Sumitomo Heavy Industries Ltd., Tokyo, Japan) with high radiochemical purity (≥ 98%). Briefly, the patient fasted for at least 6 h before examination. Chloral hydrate solution was given to the patient for sleep induction. Then, the patient received ^18^F-FDG (5.5 MBq/kg) administration intravenously and rested in a dim room for 40 min until ^18^F-FDG was distributed in the brain. Attenuation maps were generated using a CT transmission scan with a slice thickness of 3.0 mm. Then, a PET scan was performed for 10 min. PET images were reconstructed using three-dimensional ordered subsets expectation maximization (3D-OSEM) and were acquired in DICOM format using a PowerImage system (Beijing Meizhi Medical Technology Ltd., Beijing, China). In addition, image data were converted to the SUV of ^18^F-FDG using the Scenium system (Siemens Healthcare, Erlangen, Germany). Then, the mean SUV was analyzed in nine brain regions. A board-certified nuclear medicine physician reviewed the PET/CT image data and evaluated the differences before and after treatments.

### Statistical analysis

Statistical analysis was performed using SPSS software (version 20.0, IBM, Armonk, NY, USA). Data normality and homogeneity of variance were checked between groups. Primary endpoint data are presented as the mean ± SEM, and the outcome data were analyzed by two-way ANOVA with repeated measures. Additionally, the *χ*^2^ test was used for patient characteristics analysis at baseline. Statistical significance was considered if *P* < 0.05.

## Results

### Recruitment and characteristics of the patients

**Fig. 2:**
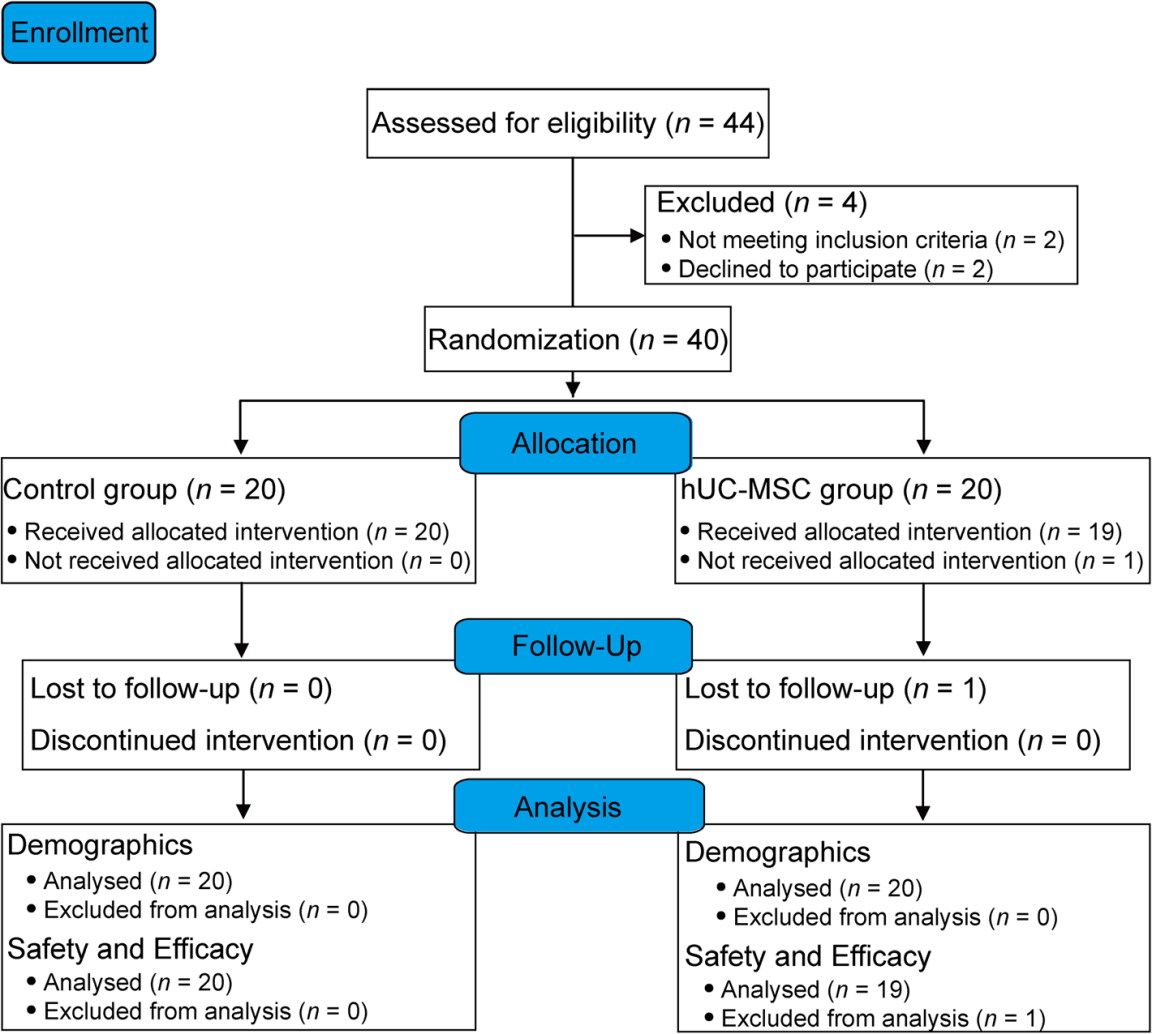
Flow diagram for patient recruitment. Forty patients were enrolled, and 39 patients completed the entire study procedure. One patient was excluded from the safety and efficacy analysis due to the withdrawal of informed consent without intervention. hUC-MSCs, human umbilical cord blood mesenchymal stem cells

In total, 40 patients were recruited from 8 August 2014 to 31 December 2016, while 1 patient withdrew informed consent without any treatment and was lost to follow-up (Fig. 2). Therefore, 39 patients completed all the study assessments. The last visit of the last patient was completed in February 2018.

With respect to the demographics of patients at baseline, no significant differences were found between groups. In particular, premature delivery and low birth weight were considered high risk factors for CP in these patients. Furthermore, the characteristics of patients were sorted out by image features of MRI. No significant difference was observed between the groups in the MRI findings (Table 2).

**Table 2:** Demographics and characteristics of patients at baseline (*n* = 40)

	All (*n* = 40)	Control (*n* = 20)	hUC-MSC (*n* = 20)	*P* value
Demographics				
Sex, *n* (%)				1.000^A^
Male	28 (70.0%)	14 (70.0%)	14 (70.0%)	
Female	12 (30.0%)	6 (30.0%)	6 (30.0%)	
Age, years, mean ± SEM	4.293 ± 0.397	4.755 ± 0.644	3.830 ± 0.459	0.249^B^
History of preterm labor, *n* (%)	16 (40.0%)	7 (35.0%)	9 (45.0%)	0.519^A^
Birth weight, kg, mean ± SEM	2.320 ± 0.042	2.395 ± 0.053	2.245 ± 0.062	0.072^B^
History of rehabilitation, *n* (%)	11 (27.5%)	6 (30.0%)	5 (25.0%)	0.723^A^
MRI findings				
Brain maldevelopments, *n* (%)	23 (57.5%)	13 (65.0%)	10 (50.0%)	0.337^A^
Cerebromalacia, *n* (%)	6 (15.0%)	3 (15.0%)	3 (15.0%)	0.658^C^
Focal ischemia, *n* (%)	1 (2.5%)	0	1 (5.0%)	1.000^D^
Corpus callosum agenesis, *n* (%)	1 (2.5%)	0	1 (5.0%)	1.000^D^
Normal, *n* (%)	9 (22.5%)	4 (20.0%)	5 (25.0%)	1.000^C^

*Abbreviations*: *hUC-MSC* human umbilical cord-derived mesenchymal stem cell, *MRI* magnetic resonance imaging

^A^*P* value was calculated by *χ*^2^ test

^B^*P* value was calculated by one-way ANOVA

^C^*P* value was calculated by correction *χ*^2^ test

^D^*P* value was calculated by Fisher’s exact test

### Safety assessments

To evaluate the safety of treatments, the AE incidence was analyzed, and no significant difference was found between groups. Evidently, upper respiratory infection was reported most frequently (61.54% of cases). Diarrhea (38.46%) and fever (28.21%) were other complaints with a high incidence (Table 3). The severity grades of AEs were mild or moderate. Serious adverse events (SAEs) were not observed. In particular, the cases occurring within 24 h of treatment were followed closely to identify graft rejection. Three cases of fever were considered to be related to the study intervention due to onset within 24 h after transplantation. In these cases, the temperature was below 38.5 °C, and all the patients recovered within 2 days after supportive treatments.

**Table 3:** Summary of adverse event in the control and the hUC-MSC groups

Group	All (*n* = 39)	Control (*n* = 20)					hUC-MSC (*n* = 19)					*P* value^A^
AE	Number (%)	Number (%)	Onset time post-treatment				Number (%)	Onset time post-treatment				
			24 h	7 days	30 days	After 30 days		24 h	7 days	30 days	After 30 days	
Upper respiratory infection	24 (61.54%)	14 (70.0%)	0	1	3	10	10 (52.6%)	0	2	2	6	0.333
Diarrhea	15 (38.46%)	9 (45.0%)	0	0	2	7	6 (31.6%)	0	0	2	4	0.514
Fever	11 (28.21%)	4 (20.0%)	0	1	1	2	7 (36.8%)	3	0	2	2	0.301
Vomiting	8 (20.51%)	3 (15.0%)	0	0	0	3	5 (26.3%)	0	0	1	4	0.451
Constipation	4 (10.26%)	3 (15.0%)	0	0	1	2	1 (5.3%)	0	0	0	1	0.605

*Abbreviations*: *AE* adverse event, *hUC-MSC* human umbilical cord-derived mesenchymal stem cell

^A^*P* value was calculated by Fisher’s exact test on the incidence between groups

### Clinical efficacy assessments

**Fig. 3:**
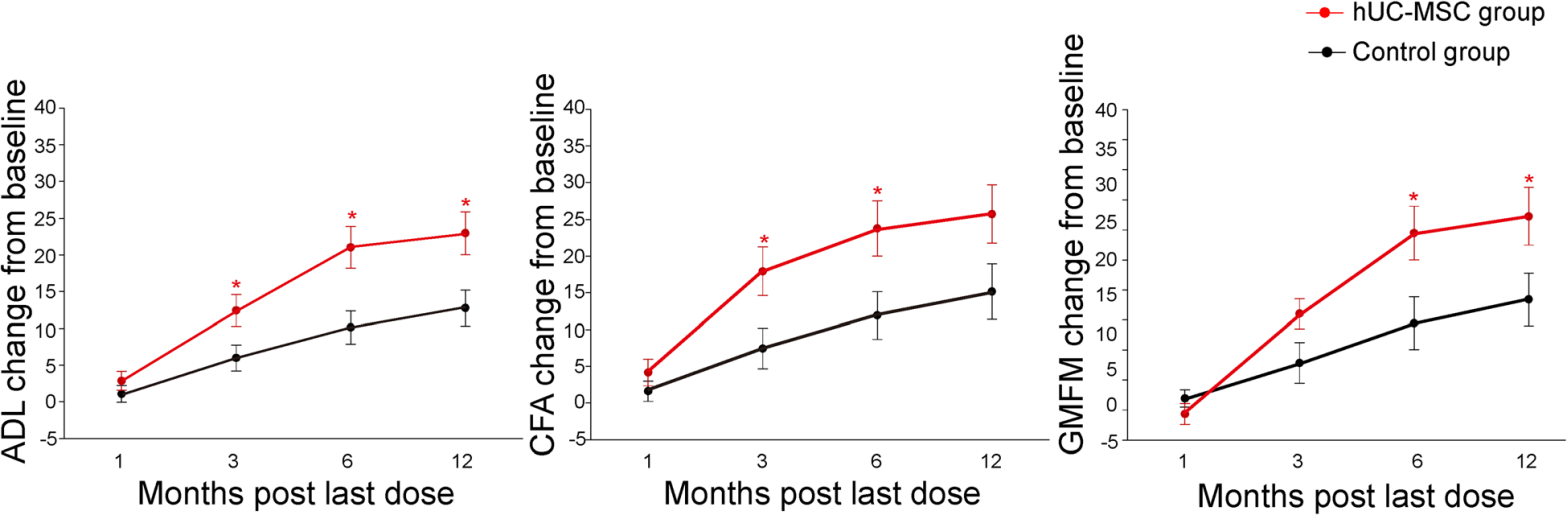
The improvements in ADL, CFA, and GMFM between groups. The improvement in scores was compared between the control group (*n* = 20) and the hUC-MSC group (*n* = 19). Improvements versus baseline at scheduled time points are presented as the mean ± SEM and were compared between groups (*P < 0.05, by two-way ANOVA with repeated measures). ADL, activities of daily living; CFA, comprehensive function assessment; GMFM, gross motor function measure

Primary endpoints were evaluated by the score changes from baseline of a series of measurable scales. There was no significant difference between groups in the ADL, CFA, and GMFM-88 scores at baseline. According to the two-way ANOVA with repeated measures, the scale scores of all functional assessments increased in both groups over time. While further comparison was performed at each time point, greater improvement in ADL scores was revealed in the hUC-MSC group at 3, 6, and 12 months after the last dose (*P* < 0.05, Table 4). Similarly, improvement in the CFA scores was superior in the hUC-MSC group at 3 and 6 months (*P* < 0.05), although it failed to reach a statistically significant difference at 12 months after the last dose (*P* = 0.061). However, greater improvement in the GMFM scores was not observed until 6 months after hUC-MSC transplantation (Fig. 3).

**Table 4:** Comparison of score changes at scheduled visits versus baseline between groups

Scale	Timeframe (month after last dose)	Control (*n* = 20), mean ± SEM	hUC-MSC (*n* = 19), mean ± SEM	*P* value^A^
ADL	1	0.658 ± 1.117	2.868 ± 1.279	0.135
	3	5.975 ± 1.782	12.447 ± 2.156	0.013*
	6	10.125 ± 2.250	21.053 ± 2.860	0.003*
	12	12.775 ± 2.465	22.974 ± 2.936	0.009*
CFA	1	1.474 ± 1.455	4.158 ± 1.804	0.324
	3	7.425 ± 2.754	17.947 ± 3.317	0.021*
	6	11.925 ± 3.253	23.790 ± 3.774	0.026*
	12	15.175 ± 3.785	25.737 ± 3.947	0.061
GMFM	1	4.053 ± 3.001	− 1.316 ± 3.429	0.237
	3	15.700 ± 6.746	32.053 ± 5.028	0.062
	6	28.900 ± 8.807	59.000 ± 8.947	0.037*
	12	36.800 ± 8.802	64.526 ± 9.600	0.045*

*Abbreviations*: *ADL* activities of daily living, *CFA* comprehensive function assessment, *GMFM* gross motor function measure, *hUC-MSC* human umbilical cord-derived mesenchymal stem cell

^A^*P* value was calculated by two-way ANOVA with repeated measures

*P < 0.05

### Cerebral metabolic changes

**Fig. 4:**
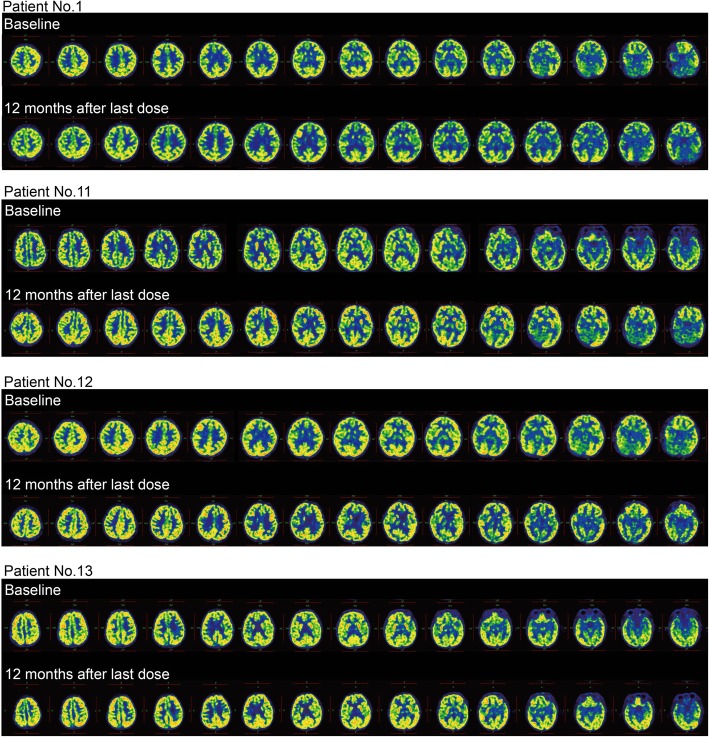
Cerebral metabolic changes after intervention. No obvious changes in the SUV were observed in the control group after intervention. However, a noticeable increase in the SUV was shown in 3 out of 5 patients from the hUC-MSC group at 12 months after the last dose (patient nos. 11, 12, and 13). SUV, standard uptake value

The SUV of ^18^F-FDG was detected by PET/CT to explore regional cerebral glucose metabolism. As an optional assessment, it was completed by 8 patients in the control group and 5 patients in the hUC-MSC group. The mean SUV was measured in cerebral regions at the last visit and was compared with the baseline level for individuals. As a result, the SUV increased over 50% in all cerebral regions of 3 patients after hUC-MSC transplantation (patient nos. 11, 12, and 13 in Table 5 and Fig. 4). In addition, no significant changes were observed in the control group.

**Table 5:** Comparison of SUV between data at 12 months after last dose and baseline

Patient no.	Basal ganglia		Central region		Cerebellum		Cingulate and paracingulate gyri		Frontal lobe		Mesial temporal lobe		Occipital lobe		Parietal lobe		Temporal lobe	
	L	R	L	R	L	R	L	R	L	R	L	R	L	R	L	R	L	R
Control group (*n* = 8)																		
1	1.12	1.56	1.03	1.16	0.90	0.82	1.11	1.25	1.17	1.08	1.00	0.67	1.00	0.99	1.12	1.16	1.24	1.03
2	1.20	1.03	0.85	0.79	0.69	0.75	0.98	0.94	1.08	1.03	0.76	0.70	0.97	0.98	0.98	0.96	0.99	0.90
3	1.15	1.14	1.23	1.12	1.16	1.11	1.04	1.06	1.06	1.08	1.13	1.08	1.09	1.02	1.16	1.05	1.10	1.05
4	0.98	0.99	0.99	0.99	0.96	0.95	1.04	1.00	1.01	0.99	0.97	0.97	1.07	1.07	1.03	1.00	1.01	0.99
5	0.86	0.95	1.09	1.13	1.26	1.25	1.15	1.17	1.20	1.24	1.10	1.11	1.14	1.17	1.23	1.18	0.99	1.06
6	1.08	1.09	1.08	1.13	1.12	1.11	1.05	1.09	1.04	1.07	1.14	1.15	1.14	1.12	1.12	1.09	1.12	1.15
7	0.95	0.97	0.97	0.98	1.09	1.11	1.06	1.04	1.01	0.98	0.97	0.93	0.93	1.01	0.98	1.05	1.06	1.06
8	0.93	0.97	1.02	1.05	1.05	1.08	1.10	1.08	1.10	1.12	0.93	1.00	1.21	1.29	1.14	1.09	1.07	1.14
hUC-MSC group (*n* = 5)																		
9	1.25	1.14	1.69	1.62	1.03	0.96	1.45	1.36	1.40	1.24	1.08	1.04	1.88	1.75	2.04	1.78	1.34	1.23
10	1.12	0.89	0.94	0.96	1.11	1.14	0.90	0.85	1.14	1.06	0.81	0.84	1.02	1.01	1.10	1.11	1.10	0.87
11	2.97	3.66	3.35	2.94	3.27	3.08	2.78	2.60	3.68	3.94	1.71	3.07	4.83	5.62	2.81	4.19	3.75	4.25
12	1.59	1.70	1.73	1.77	1.72	1.65	1.73	1.68	1.73	1.76	1.73	1.75	1.89	1.83	1.81	1.73	1.83	1.79
13	6.91	6.44	6.72	7.71	4.88	5.21	6.45	6.72	6.99	7.24	4.38	3.05	7.62	7.58	7.87	6.77	8.98	8.36

Data was calculated with SUV2 divided by SUV1. SUV1 referred to mean SUV at baseline. SUV2 referred to mean SUV at 12 months after last dose

*Abbreviations*: *hUC-MSC* human umbilical cord-derived mesenchymal stem cell

## Discussion

Our clinical findings indicate that hUC-MSC transplantation significantly enhances gross motor and cognitive functions on the basis of rehabilitation in children with CP. There is increasing evidence showing that hUC-MSCs have therapeutic potential for CP in clinical studies. Promising safety and efficacy data were presented in several case reports regarding hUC-MSCs concomitant with [30] or without [31, 32] rehabilitation. However, few randomized trials focused on hUC-MSC transplantation intravenously concomitant with rehabilitation. Based on our preliminary safety and efficacy data in a case study [33] and a single-blinded trial [34], we conducted this double-blinded clinical trial to further suggest the therapeutic benefits of hUC-MSC transplantation in patients with CP and proposed the possible mechanism of enhancing brain function using ^18^F-FDG-PET/CT detection.

With respect to the safety of hUC-MSC transplantation, favorable results were shown up to 12 months after treatment. No significant difference was found between groups in AE incidence. No SAE was observed during the entire study. It was also noteworthy that three cases of transient slight fever were considered to be related to hUC-MSC transplantation. The incidence of fever in the hUC-MSC group during the study (36.8%) was similar to prior reports with [35] or without immunosuppressant pre-treatment [36]. There was some evidence to suggest that hUC-MSCs were immunologically safe for clinical application, owing to their low expression of major histocompatibility complex class I (MHC-I) and MHC-II [37]. In addition to low immunogenicity, their immunomodulatory effects were also attributed to immunological safety for clinical application [38], which was supported by the benefits of hUC-MSCs against chronic graft-versus-host disease after HLA-haploidentical stem cell transplantation [39]. Therefore, HLA disparities were not tested based on the immunoprivileged features of hUC-MSCs shown in previous studies. In addition, immunosuppressants were not adopted in our study, balancing the risk and benefit of patients. Concerning long-term safety, data were still collected continually in our extended follow-up program upon patients’ agreements, although encouraging safety data have been shown in patients with systemic lupus erythematosus [40] and patients with osteoarthritis receiving hUC-MSC transplantation [41].

Regarding the therapeutic efficacy, our ADL, CFA, and GMFM assessment data indicated that the therapeutic effects of rehabilitation were enhanced significantly by hUC-MSC transplantation for both gross motor and cognitive functions. This finding was consistent with that in the UCB transfusion study [35]. We also noticed that the improvements reached a peak at 6 months and lasted up to 12 months after hUC-MSC transplantation. The long-lasting positive influence could be attributed to the paracrine effects of hUC-MSCs according to the evidence in a preclinical study [42]. It is a commonly held belief that the treatment efficacy is closely related to the therapeutic window, transplantation route, and dosage. The therapeutic window of cell therapy was suggested within 72 h postnatal for infants with hypoxic-ischemia encephalopathy [43]. However, patients with CP usually could not be diagnosed until several years after brain injury. In this phase, therapeutic benefits are quite limited from routine therapies, although motor function could be improved partly by rehabilitation [44]. Nonetheless, there are still hopes for CP treatment to alleviate the persistent inflammation and gliosis in the tertiary phase of brain damage [45]. Our data suggest that the patients still benefit from hUC-MSC transplantation in a therapeutic window extending to years after brain injury. The improvements were likely related to the anti-inflammatory potential of hUC-MSCs since IL-1α, IL-6, and TNF-β were decreased significantly after transplantation [46]. When considering the transplantation route of cell therapy, intravenous administration was preferred to intrathecal injection for better compliance, since pain at the injection site was a complaint due to intrathecal administration [16]. In addition, hUC-MSCs were given at dosages of 4.5~5.5 × 10^7^ cells intravenously in our study, which was supported by the suggested doses in the Clinical Cell Therapy Guidelines for Neurorestoration [47]. Moreover, repeated dosing was adopted instead of high-dosage single administration to reduce the risk of cell embolism in our study. Currently, the transplantation dosage varied greatly (5~50 × 10^6^ cells per dose) with different transplantation routes (intrathecal injection, intravenous transfusion, or both) in case studies [31–33]. These potential biases might lead to inconclusive evaluation of the benefit of hUC-MSC transplantation. The therapeutic window, transplantation route, and dosage in our trial were considerable for a systemic evaluation in future studies.

In terms of the pathogenesis of CP, lower regional cerebral glucose metabolism was detected by PET in an asphyxiated infant developing CP [48]. Therefore, cerebral metabolic activity was evaluated by the SUV of ^18^F-FDG in our study to explore the mechanism of functional improvements after hUC-MSC transplantation. A noticeable increase in the SUV was observed in three out of five patients from the hUC-MSC group, while no significant changes were observed in the control group. This finding suggested that the amelioration of cerebral metabolism might play an essential role in the functional improvement induced by hUC-MSC transplantation. And it was consistent with a previous study on UCB infusion in patients with CP [35]. Additionally, decreased periventricular inflammation was observed by PET scans in patients after UCB infusion, while plasma levels of pentraxin 3 and interleukin-8 were indicated to be related to motor function improvements [49]. However, it was still unclear whether the improvement in cerebral metabolism was partially attributed to the anti-inflammatory potential of hUC-MSCs. Further information in this field would help us move forward.

Potential limitations existed in this study. First, the small sample size did not allow for stratified analysis of the age range and neuroimaging features of patients. Thus, the individual discrepancy in therapeutic efficacy could not be addressed in this study. Second, cerebral metabolic activity could not be analyzed between groups due to data limitation. More evidence was needed to identify the correlation between cerebral metabolism and functional improvement after hUC-MSC transplantation. Finally, the study was limited by a lack of information necessary for mechanism explanation. Further research on the plasma level of inflammatory cytokines before and after intervention would be worthwhile in future in-depth explorations.

## Conclusion

The current study evaluated the efficacy and safety of hUC-MSC transplantation in patients with CP. Our data suggested that hUC-MSC transplantation with basic rehabilitation was safe and more effective at improving gross motor and cognitive function in children with CP. The findings provide insights into a novel therapeutic strategy for patients with CP. The therapeutic window, transplantation route, and dosage in our study were considerable for clinical application. Notwithstanding the relatively limited samples, our exploration of cerebral metabolic activity offered some insights into the mechanism of hUC-MSC treatments. Further study could focus on neuroimaging and neuroinflammation to elucidate the exact mechanism. In addition, it was helpful for clinical translation to identify precise patient populations by stratified analysis. More safety data are needed to evaluate the long-term benefit of hUC-MSC transplantation.

## Data Availability

Individual participant data that underlie the results reported in this article are available after deidentification for investigational purpose, beginning 9 months and ending 5 years following article publication. Data are available in our hospital’s clinical trial medical records managed by GCP office with investigator’s support. The study protocol could be available with registration number as ChiCTR1800016554 and public title as “Randomized trial of umbilical cord-derived mesenchymal stem cells for cerebral palsy” (http://www.chictr.org.cn/showproj.aspx?proj=27139).

## Abbreviations

^18^F-FDG-PET/CT: ^18^F-fluorodeoxyglucose positron emission tomography/computed tomography
ADL: Activities of daily living
AE: Adverse event
CFA: Comprehensive function assessment
CP: Cerebral palsy
GMFM: Gross motor function measure
hUC-MSCs: Human umbilical cord mesenchymal stem cells
IRB: Institutional review board
MRI: Magnetic resonance imaging
NS: Normal saline
SAE: Serious adverse event
SUV: Standard uptake value
UCB: Umbilical cord blood
